# Elevated neutrophil-to-lymphocyte ratios correlate with increased clozapine concentration-to-dose ratios during titration

**DOI:** 10.1038/s41537-025-00648-4

**Published:** 2025-07-10

**Authors:** Bunichiro Onodera, Mutsumi Sakata, Kazuro Ikawa, Daisuke Kume, Naoki Horikawa, Hiroshi Komatsu, Takuhiro Yamaguchi, Hiroaki Tomita, Yuki Kikuchi

**Affiliations:** 1 Department of Psychiatry, Graduate School of Medicine, Tohoku University, Sendai, Miyagi Japan; 2 Department of Psychiatry, Kodama Hospital, Ishinomaki, Miyagi Japan; 3 Nozoe Hills Hospital, Fukuoka, Japan; 4 Department of Clinical Pharmacotherapy, Hiroshima University, Hiroshima, Japan; 5 Department of Psychiatry, Tohoku University Hospital, Sendai, Miyagi Japan; 6 Division of Biostatistics, Tohoku University Graduate School of Medicine, Sendai, Japan

**Keywords:** Biomarkers, Pharmacology

## Abstract

Few cohort studies have examined the relationship between inflammation and increased clozapine blood levels. The purpose of this study was to investigate the relationship between the neutrophil-to-lymphocyte ratio (NLR), a marker of inflammation, and the clozapine concentration-to-dose (C/D) ratio during clozapine titration. We retrospectively investigated the medical records of all patients at Nozoe Hills Hospital who met the following criteria: 1) patients with schizophrenia who were first treated with clozapine between April 2020 and July 2024 and 2) patients for whom clozapine blood levels were measured for at least two consecutive weeks after the start of clozapine treatment. The study included 143 blood samples from 28 patients collected within 6 weeks of starting clozapine treatment. A linear mixed model with random intercepts was used to determine the correlation between the clozapine C/D ratio and NLR in samples repeatedly measured within an individual. Fixed effects for the C/D ratio included NLR, week, and the interaction between NLR and week. A significant fixed effect of NLR on C/D ratio was observed (estimate: 0.70; 95% confidence interval: 0.47–0.92; *P* < 0.0001). The fixed effect of NLR was attenuated over time due to a significant negative interaction between NLR and week. The fixed effect of NLR remained significant even after excluding the six patients who had fever during clozapine titration. This study suggests a positive correlation between the C/D ratio and NLR during clozapine titration. Our findings indicate that subclinical inflammation in the early titration phase affects the pharmacokinetics of clozapine.

## Introduction

Clozapine is a highly effective atypical antipsychotic used for the management of treatment-resistant schizophrenia; however, it is underused because of its various side effects. The ability to metabolize clozapine varies significantly among individuals^1,2^. Factors such as sex, smoking, and ethnicity are known to affect clozapine metabolism, and international guidelines recommend initial titration protocols that consider these factors^1^. Rapid clozapine titration that is not appropriate for an individual’s metabolic capacity can lead to early inflammatory side effects such as fever, myocarditis, and pneumonia^3–6^. This may progress to a life-threatening condition characterized by organ damage such as myocarditis, pneumonia, nephritis, liver dysfunction, and pancreatitis, which may be accompanied by eosinophilia and rash, and is diagnosed as clozapine-associated drug reaction with eosinophilia and systemic symptoms (DRESS) syndrome^7–9^. These conditions are thought to be caused by an immune hypersensitivity reaction to clozapine. The relative mortality rate of myocarditis and pneumonia associated with clozapine is significantly higher than that of clozapine-associated agranulocytosis^10,11^. Therefore, it is important to estimate the clozapine-metabolizing ability of an individual early in the titration phase and perform individualized titration. Measuring the clozapine concentration-to-dose (C/D) ratio helps estimate the individual metabolic capacity. Our previous study analyzing the weekly measurements of clozapine blood concentrations within 6 weeks of starting clozapine revealed that fever increased the clozapine C/D ratio^12^. Inflammation reportedly reduces the activity of cytochrome P450 (CYP) 1A2, the main enzyme involved in the metabolism of clozapine^13^. The underlying mechanism is hypothesized to be that inflammatory cytokines such as interleukin-6 and tumor necrosis factor-α suppress the transcription of CYP1A2, thereby reducing its synthesis and activity^14,15^. The inflammation induced by clozapine itself may impair its metabolism, leading to increased clozapine blood levels and further inflammation, creating a vicious cycle^16^. Therefore, international guidelines recommend measuring C-reactive protein (CRP) weekly for the first 4 weeks after initiating clozapine and avoiding dose increases if CRP levels rise^1^. Our previous study measured CRP levels weekly or daily during clozapine titration and reported that CRP levels were elevated in many patients even in the absence of obvious inflammatory symptoms such as fever^17^. Furthermore, we showed that the C/D ratio may temporarily increase even in patients without fever^12^. Based on these results, we hypothesized that subclinical inflammation during clozapine titration increases the clozapine C/D ratio. Most of the data on the relationship between inflammation and elevated blood clozapine levels is based on case reports, and studies with well-controlled cohorts are lacking^18,19^. A recent retrospective study by Smith et al. identified 126 patients who had their clozapine blood levels and CRP levels measured simultaneously, demonstrating an association between CRP and clozapine blood levels^20^. However, they were limited as the clinical context of each patient and the timing of the blood measurements (number of days after starting clozapine) were unknown, and the longitudinal change in the C/D ratio within patients was unknown^21^. Therefore, we decided to examine the relationship between inflammation and clozapine blood levels using past datasets. Although CRP was not measured simultaneously with clozapine blood levels, neutrophil and lymphocyte counts were routinely measured in clinical practice. We therefore decided to use the neutrophil/lymphocyte ratio (NLR) as a marker of inflammation.

NLR is a readily available, robust biomarker of inflammation, stress, and immune system activation that was proposed by Zahorec more than 20 years ago^22,23^. NLR is widely used in general internal medicine (inflammation and infection), intensive care medicine, oncology, surgery, and perioperative medicine, and has been established as a very sensitive marker of inflammation and stress^23^. It is also used to assess inflammation in chronic diseases, including diabetes, cancer, ischemic heart disease, and psychiatric disorders^23^. There is growing evidence suggesting that inflammation may be involved in the pathogenesis and prognosis of psychiatric disorders^24,25^ and NLR is extensively used as an indicator of inflammation in psychiatric research^26^.

Thus, the present study aimed to investigate whether an increase in NLR, an alternative marker of inflammation^22,23^, would be associated with an increase in the C/D ratio upon reanalyzing the dataset of our previous publication^12^, in which the blood concentration of clozapine and the number of neutrophils and lymphocytes were measured simultaneously every week for 6 weeks from the start of treatment.

## Methods

### Study participants

The present study is a reanalysis of data from our previous publication, in which clozapine levels were measured weekly for 6 weeks after starting treatment^12^. This study is a secondary analysis of this dataset. Briefly, we retrospectively investigated the medical records of all patients at Nozoe Hills Hospital who met the following criteria: 1) patients with schizophrenia who were first treated with clozapine between April 2020 and July 2024 and 2) patients for whom clozapine blood levels were measured for at least two consecutive weeks after the start of clozapine treatment. Schizophrenia was diagnosed based on the International Classification of Diseases, 10th revision. Because this retrospective study used anonymized data, an opt-out form was displayed on the hospital’s bulletin board before data collection. Individuals who did not express their intent for exclusion were included in the study. This study was approved by the Ethics Committee of Nozoe Hills Hospital (Approval ID: 2024-6).

### Measurement of clozapine blood levels

All blood concentration measurements were performed in an inpatient setting and the vital signs and medication adherence of the patients were monitored by a nurse. Smoking was prohibited at the hospital. The details of concomitant medications at the start of clozapine treatment have been described previously^12^, and there was no concomitant use of potent CYP1A2 inhibitors. Blood clozapine levels within 6 weeks of starting clozapine treatment were included in this study. Clozapine levels were measured simultaneously with weekly blood tests for white blood cell and neutrophil counts, as required in Japan, after starting clozapine. In most patients, blood was collected ~12 h after the last clozapine dose. Blood clozapine levels were measured by high-performance liquid chromatography at the Department of Clinical Pharmacotherapy, Hiroshima University, Hiroshima, Japan, as previously reported^12^. Blood clozapine levels measured while the temperature of the patient exceeded 38 °C until it dropped below 37 °C were classified as measurements taken during fever.

### Assessment of C/D ratio and NLR

The average clozapine dose up to 5 days before the blood clozapine level measurements was used to calculate the C/D ratios for each patient. This is because the clozapine dose changed during titration, and steady-state blood concentration measurements were not available. Generally, ~95% of the steady-state concentration is achieved in five half-lives, and 99% is achieved in seven half-lives^27^. Assuming a half-life of 24 h for repeated doses of clozapine, a steady state can be achieved in five days^28^.

The NLR or neutrophil counts/lymphocyte counts were calculated simultaneously with the blood clozapine concentration measurement.

### Statistical analysis

Statistical analyses were performed using the EZR software (Jichi Medical University, Saitama, Japan). First, to roughly estimate the overall correlation, Pearson’s product-moment correlation coefficient was calculated for all samples for the correlation between C/D ratio and NLR, C/D ratio and neutrophil counts, and C/D ratio and lymphocyte counts. Next, a linear mixed model with restricted maximum likelihood estimation was used to clarify the correlation between the C/D ratio and NLR in samples that were repeatedly measured within individuals. The fixed effects used to estimate the C/D ratio included the NLR (continuous variable), week (continuous variable), and the interaction between the NLR and week. We also analyzed sex, obesity (body mass index [BMI] >30), concomitant valproate use, and smoking before hospitalization as covariates in a linear mixed model as a sensitivity analysis. The random effects considered the individual variability of the intercept. Because the C/D ratio and NLR were not normally distributed, they were transformed to the natural logarithm. We performed a sensitivity analysis, excluding six patients who developed fever within 6 weeks after starting clozapine treatment. The statistical significance level was set at *P* < 0.05.

## Results

A total of 28 patients were included in the study. There were 143 clozapine blood samples measured weekly within 6 weeks of clozapine initiation. Detailed patient demographics and clozapine blood concentration data are shown in Table 1. Of the 28 patients, six had fever within 6 weeks of clozapine initiation, but only three blood clozapine samples were measured during fever. In all 143 samples, the median C/D ratio (interquartile range [IQR]) was 1.43 (1.05–2.0), and the median NLR (IQR) was 1.85 (1.43–2.79). Table 2 shows ranges of C/D ratios and NLRs by week. Although we have previously reported weekly C/D ratios using the same cohort^12^, the present study describes a more detailed range. From weeks 1–3, the median NLR was significantly higher in patients with fever than in those without (Table 2). As we reported previously^12^, in weeks 1 and 2, the median C/D ratio was significantly higher in patients with fever than in those without (Table 2).

**Table 1:** Patient characteristics and blood clozapine measurements.

Number of patients	28
Female, *n* (%)	15 (54)
Age, year, mean (SD)	30.6 (16.1)
BMI, kg/m^2^, mean (SD)	23.3 (5.2)
Obesity (BMI > 30), *n* (%)	3 (11)
Smoking before starting clozapine, *n* (%)^a^	4 (14)
Concomitant valproate at clozapine initiation, *n* (%)	8 (29)
Body temperature before starting clozapine, °C, median (min, IQR, max)	36.4 (35.3, 36.2–36.6, 37.1)
Patient with fever, *n* (%)	6 (21)
Number of clozapine blood levels measured, n	143
Number of measurements during fever, n (%)	3 (2.1)
CRP levels measured during fever, mg/dL, mean (SD) (*n* = 5)	8.61 (4.85)
Clozapine dose, mg/day, median (min, IQR, max)	75 (25, 45–100, 300)
Clozapine blood levels, ng/mL, median (min, IQR, max)	93 (13, 57–153.5, 626)
C/D ratio, (ng/mL)/(mg/day), median (min, IQR, max)	1.43 (0.26, 1.05–2.0, 6.96)
White blood cell count, ×10^3^/mm^3^, median (min, IQR, max)	6.02 (4.0, 5.20–7.68, 17.8)
Neutrophil count, ×10^3^/mm^3^, median, (min, IQR, max)	3.42 (2.01, 2.61–4.65, 12.0)
Lymphocyte count, ×10^3^/mm^3^, median (min, IQR, max)	1.91 (0.42, 1.43–2.33, 4.61)
NLR, median (min, IQR, max)	1.85 (0.75, 1.42–2.79, 9.14)

*BMI* body mass index, *C/D* concentration-to-dose, *CRP* C-reactive protein, *IQR* interquartile range, *NLR* neutrophil-to-lymphocyte ratio, *min* minimum, *max* maximum.

^a^Smoking was prohibited during clozapine titration.

**Table 2:** Ranges of weekly C/D ratios and NLRs.

	C/D ratio, median (min, IQR, max)			NLR, median (min, IQR, max)		
	Patients with fever	Patients without fever	*P*^a^	Patients with fever	Patients without fever	*P*^a^
Week 1 (*n* = 28)	2.12 (1.43, 1.87–3.06, 4.49)	1.31 (0.35, 0.94–1.88, 2.80)	0.022	3.16 (1.46, 2.32–4.53, 9.14)	1.31 (0.79, 1.14–1.56, 2.98)	<0.001
Week 2 (*n* = 28)	3.48 (1.68, 2.30–4.18, 6.96)	1.23 (0.35, 0.94–2.12, 2.82)	0.01	3.50 (2.04, 3.36–4.50, 7.36)	1.76 (0.75, 1.48–2.33, 5.34)	<0.001
Week 3 (*n* = 25)	2.80 (1.20, 1.60–4.20, 5.20)	1.32 (0.55, 1.14–1.67, 4.10)	0.11	4.57 (2.95, 3.74–4.57, 6.49)	1.86 (0.93, 1.21–2.22, 7.96)	0.0085
Week 4 (*n* = 27)	1.12 (0.94, 1.07–1.83, 2.59)	1.33 (0.26, 1.03–1.73, 3.42)	0.93	3.17 (1.61, 3.14–5.86, 5.88)	1.89 (0.95, 1.45–2.45, 7.89)	0.055
Week 5 (*n* = 18)	2.01 (1.12, 1.75–2.10, 2.27)	1.33 (0.43, 1.18–2.07, 2.76)	0.57	2.28 (1.61, 1.69–3.47, 5.38)	1.97 (0.94, 1.48–2.47, 4.95)	0.442
Week 6 (*n* = 17)	1.49 (1.26, 1.38–2.12, 2.74)	1.32 (0.28, 1.02–1.59, 2.64)	0.31	1.87 (1.49, 1.68–3.44, 5.00)	1.61 (0.85, 1.20–3.31, 7.49)	0.59

*C/D* concentration-to-dose, *IQR* interquartile range, *NLR* neutrophil-to-lymphocyte ratio, *min* minimum, *max* maximum

^a^The weekly C/D ratios and NLRs were compared between patients with and without fever using the Mann–Whitney *U* test.

**Fig. 1:**
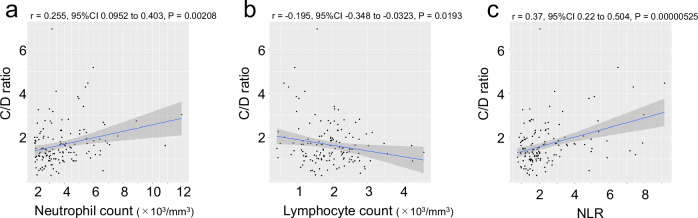
Pearson's product-moment correlation analysis. **a** correlation coefficient between the C/D ratio and neutrophil count. **b** correlation coefficient between the C/D ratio and lymphocyte count. **c** correlation coefficient between the C/D ratio and NLR. The shaded area indicates the confidence interval at the 95% confidence level. *C/D ratio* concentration-to-dose ratio, *NLR* neutrophil-to-lymphocyte ratio.

Pearson’s product-moment correlation analysis indicated a significant correlation between the C/D ratio and neutrophil count (correlation coefficient = 0.255, 95% confidence interval [CI]: 0.0952–0.403, *P* < 0.005), as shown in Fig. 1a. In contrast, a significant negative correlation was shown between the C/D ratio and lymphocyte count (correlation coefficient = −0.195, 95% CI: −0.348 to −0.0323, *P* < 0.05) (Fig. 1b). Among these analyses, the correlation between the C/D ratio and NLR was the strongest (correlation coefficient = 0.37, 95% CI: 0.22–0.504, *P* < 0.00001) (Fig. 1c).

**Fig. 2:**
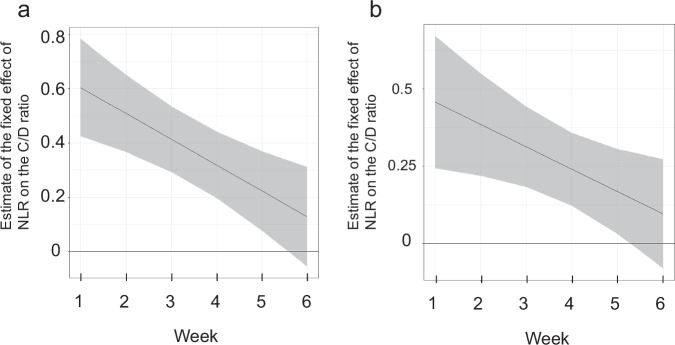
Estimates of the fixed effects of the NLR by week on the C/D ratio. **a** Analysis of all patients (28 patients, 143 samples). **b** Sensitivity analysis excluding the six patients who had fever within 6 weeks after starting clozapine treatment (22 patients, 115 samples). The shaded area indicates the confidence interval at the 95% confidence level. *C/D ratio* concentration-to-dose ratio, *NLR* neutrophil-to-lymphocyte ratio.

Next, we used a linear mixed model to examine the fixed effect of NLR on the C/D ratio in repeatedly measured samples within individuals. A significant fixed effect of NLR on C/D ratio was observed (estimate: 0.70; 95% CI: 0.47 to 0.92; *P* < 0.0001) (Table 3). Since we found significant interactions between NLR and week, we calculated an estimate of the fixed effect of NLR on the C/D ratio with the week interaction considered (Fig. 2a). The fixed effect of NLR was attenuated over time due to a significant negative interaction between NLR and week. (Fig. 2a). As a sensitivity analysis, the same analysis was performed excluding the six patients who had fever within 6 weeks after starting clozapine treatment. The results of the sensitivity analysis were generally consistent with those of the main analysis (Table 4, Fig. 2b). We also performed a sensitivity analysis by including sex, obesity (BMI > 30), concomitant use of valproic acid, and smoking before hospitalization as covariates in a linear mixed model, but no significant effect was observed for any of these factors (Supplementary Tables 1–4).

**Table 3:** Estimates of fixed effects on the C/D ratio in a linear mixed model (all patients).

			95% confidence interval			
	Estimate	SE	Lower limit	Upper limit	T value	P
NLR	0.70	0.12	0.47	0.92	6.13	<0.0001
Week	0.02	0.02	−0.03	0.06	0.69	0.493
NLR × Week	−0.10	0.03	−0.15	−0.04	−3.38	<0.001

Note: Number of patients = 28; Number of blood samples = 143.

The C/D ratio and NLR were converted to natural logarithms.

*C/D* concentration-to-dose, *NLR* neutrophil-to-lymphocyte ratio, *SE* standard error.

**Table 4:** Estimates of fixed effects on the C/D ratio in a linear mixed model (excluding the six patients with fever).

			95% CI			
	Estimate	SE	Lower limit	Upper limit	*T* value	*P*
NLR	0.53	0.14	0.26	0.79	3.89	<0.001
Week	0.02	0.02	−0.02	0.06	0.84	0.40
NLR × Week	−0.07	0.03	−0.14	−0.01	−2.29	0.024

Note: Number of patients = 22; Number of blood samples = 115

The C/D ratio and NLR were converted to natural logarithms.

*C/D* concentration-to-dose, *NLR* neutrophil-to-lymphocyte ratio, *SE* standard error.

## Discussion

The present study’s main finding was identifying a positive correlation between NLR and C/D ratio during clozapine titration. Although the relationship between inflammation and increased blood clozapine levels has been demonstrated in numerous case reports^18^, only a few cohort studies have been conducted under well-controlled conditions^19^. In a cohort of 131 Chinese patients^29^, inflammation/infection was observed on 2% of the total monitored days (482/24,789), occurring in 18 episodes across 16 patients. Eleven percent of the infection episodes did not show any clinically relevant effect (no increase in white blood cells or CRP levels). In 61% of the infection episodes, it was recommended to reduce the clozapine dose by half, and in 28% of the infection episodes, it was recommended to reduce the clozapine dose by one-third. In a cohort of 65 hospitalized Chinese patients^15^, the clozapine C/D ratios during infection were significantly higher than at baseline, and a significant positive correlation was found between the C/D ratios and white blood cell and neutrophil counts. Recently, Smith et al. showed in a retrospective study of 126 patients that the C/D ratio was three times higher in non-smokers with high CRP levels (CRP > 50 mg/L) than in those with low CRP levels (CRP < 5 mg/L)^20^.

The strength of the current study is that blood clozapine levels and NLR were measured under well-controlled conditions every week for 6 weeks after clozapine initiation during hospitalization. Furthermore, even when the six patients who had fever during clozapine titration were excluded, a significant correlation was observed between the clozapine C/D ratio and the NLR, suggesting that blood clozapine levels increased when the NLR, a marker of inflammation, was high, even when the patient was clinically asymptomatic. The reference range for NLR is 1 to 2, and values higher than 3 are considered pathological^30^. The linear mixed model of this study showed that when the NLR increased by 1%, the C/D ratio increased by 0.70%. In weeks 1–3, the median NLR was significantly higher in patients with fever than in those without, exceeding 3, and the median C/D ratio was also significantly higher in patients with fever than in those without in weeks 1 and 2 (Table 2). Therefore, although no clear threshold for NLR was identified in the present study, the possibility of unexpectedly high blood levels of clozapine may be considered when the NLR exceeds 3.

In addition, the present study showed that the fixed effect of NLR on the C/D ratio significantly decreased over time. This fact may suggest that subclinical inflammation is likely to occur early after the start of clozapine (weeks 1–3), i.e., the NLR and C/D ratio are likely to increase at this time. However, the frequency and onset time of inflammation may be affected by titration speed^4,31,32^.

Our results suggest that subclinical inflammation during clozapine titration can increase blood clozapine levels more than expected and underscore the importance of CRP monitoring in detecting subclinical inflammation during clozapine titration. As international guidelines recommend measuring CRP and clozapine blood levels weekly after starting clozapine^1^, clinicians should monitor CRP and clozapine blood levels^33^. CRP levels were markedly higher than the reference range in all samples during fever (Table 1; *n* = 5). Considering that the median NLR was significantly higher in patients with fever than in those without (Table 2), CRP, NLR, and C/D ratio may be correlated. However, although CRP and NLR are both inflammatory biomarkers, there may be differences in the timing and degree of elevation between CRP and NLR, and further studies are needed regarding their clinical applicability.

The results of this study show that clinicians can estimate inflammation and elevated clozapine blood levels using neutrophil and lymphocyte counts, which are routinely measured in clinical practice during clozapine titration. By monitoring CRP levels and NLR during clozapine titration, clinicians can detect subclinical inflammation early and take measures such as temporarily halting titration, thereby preventing the progression of clozapine-induced life-threatening conditions such as myocarditis, pneumonia, and DRESS syndrome.

In this cohort, the median clozapine dose at week 4 was 75 mg/day, and the median clozapine blood level at week 4 was 116 ng/mL. In a study of 1408 Korean inpatients, the average clozapine dose was ~200 mg/day after 4–8 weeks of initiation, and the average C/D ratio was 1.23 ng/mL per mg/day^34^. International guidelines recommend the slowest titration for non-smoking Asian women, with 175 mg/day for normal metabolizers and 75 mg/day for poor metabolizers at week 4 after starting clozapine^35^. Naturalistic observations may indicate that Asian populations tolerate clozapine at lower doses than European populations.

This study has several limitations. First, the number of patients included was small. Further studies with larger sample sizes are needed to confirm our results. Second, it is difficult to use steady-state concentrations during titration; therefore, the average dose over the five days before measuring blood concentration was used to calculate the C/D ratio. However, since the participants in the present study were titrated very slowly (Table 1)^12^, the clozapine dose remained almost unchanged over 5 days, and this limitation had little effect on our findings. Finally, this study was conducted entirely on Japanese patients; therefore, while it has the advantage of being a homogeneous ethnic group, it cannot be generalized to other populations. Japanese people belong to the East Asian ethnic group and have the lowest clozapine metabolism capacity among all ethnic groups^1,2^. International guidelines recommend the slowest titration speed for Japanese people^1^. The frequency of inflammatory side effects caused by clozapine is reported to be higher in Japanese people than in Europeans^3^. It cannot be ruled out that both clozapine metabolism capacity and immunological responsiveness to clozapine differ among ethnic groups. Therefore, future studies should clarify the relationship between clozapine titration and inflammation in other ethnic groups.

In conclusion, this study suggests a positive correlation between the C/D ratio and NLR during clozapine titration. Our findings indicate that subclinical inflammation in the early titration phase affects the pharmacokinetics of clozapine.

## Supplementary information

Supplementary Tables 1–4

## Data Availability

The data are not publicly available because they contain information that could compromise the research participants’ privacy/consent.
