# Supplementary material for: Elevated neutrophil-to-lymphocyte ratios correlate with increased clozapine concentration-to-dose ratios during titration
Source: Schizophrenia (Heidelb). 2025 Jul 10;11(1):96. doi: 10.1038/s41537-025-00648-4 (PMC12246045; doi:10.1038/s41537-025-00648-4)
Supplement: Supplementary file 1 — Supplementary Tables 1–4 [file 41537_2025_648_MOESM1_ESM.docx]

| Supplementary Table 1: Estimates of fixed effects on the C/D ratio in a linear mixed model (sensitivity analysis of fixed effects of sex) | | | | | | |
| --- | --- | --- | --- | --- | --- | --- |
|  |  |  | 95% confidence interval | |  |  |
|  | Estimate | SE | Lower limit | Upper limit | T value | P |
| NLR | 0.70 | 0.14 | 0.48 | 0.92 | 6.12 | < 0.0001 |
| Week | 0.02 | 0.02 | −0.03 | 0.06 | 0.69 | 0.49 |
| Sex (Male) | −0.05 | 0.17 | −0.39 | 0.28 | −0.32 | 0.75 |
| NLR × Week | −0.09 | 0.03 | −0.15 | −0.04 | −3.38 | < 0.001 |
| Note: Number of patients = 28; Number of blood samples = 143 | | | | | | |
| The C/D ratio and NLR were converted to natural logarithms. | | | | | | |
| C/D, concentration-to-dose; NLR, neutrophil-to-lymphocyte ratio; SE, standard error | | | | | | |

| Supplementary Table 2: Estimates of fixed effects on the C/D ratio in a linear mixed model (sensitivity analysis of fixed effects of concomitant valproate) | | | | | | |
| --- | --- | --- | --- | --- | --- | --- |
|  |  |  | 95% confidence interval | |  |  |
|  | Estimate | SE | Lower limit | Upper limit | T value | P |
| NLR | 0.70 | 0.07 | 0.48 | 0.92 | 6.13 | < 0.0001 |
| Week | 0.02 | 0.02 | −0.03 | 0.06 | 0.69 | 0.49 |
| Concomitant valproate | 0.04 | 0.19 | −0.33 | 0.41 | 0.20 | 0.85 |
| NLR × Week | −0.10 | 0.03 | −0.15 | −0.04 | −3.38 | < 0.001 |
| Note: Number of patients = 28; Number of blood samples = 143 | | | | | | |
| The C/D ratio and NLR were converted to natural logarithms. | | | | | | |
| C/D, concentration-to-dose; NLR, neutrophil-to-lymphocyte ratio; SE, standard error | | | | | | |

| Supplementary Table 3: Estimates of fixed effects on the C/D ratio in a linear mixed model (sensitivity analysis of fixed effects of obesity) | | | | | | |
| --- | --- | --- | --- | --- | --- | --- |
|  |  |  | 95% confidence interval | |  |  |
|  | Estimate | SE | Lower limit | Upper limit | T value | P |
| NLR | 0.70 | 0.11 | 0.48 | 0.92 | 6.13 | < 0.0001 |
| Week | 0.02 | 0.02 | −0.03 | 0.06 | 0.69 | 0.49 |
| Obesity (BMI>30) | 0.04 | 0.28 | −0.50 | 0.58 | 0.14 | 0.89 |
| NLR × Week | −0.10 | 0.03 | −0.15 | −0.04 | −3.38 | < 0.001 |
| Note: Number of patients = 28; Number of blood samples = 143 | | | | | | |
| The C/D ratio and NLR were converted to natural logarithms. | | | | | | |
| BMI, body mass index; C/D, concentration-to-dose; NLR, neutrophil-to-lymphocyte ratio; SE, standard error | | | | | | |

| Supplementary Table 4: Estimates of fixed effects on the C/D ratio in a linear mixed model (sensitivity analysis of fixed effects of smoking before hospitalization) | | | | | | |
| --- | --- | --- | --- | --- | --- | --- |
|  |  |  | 95% confidence interval | |  |  |
|  | Estimate | SE | Lower limit | Upper limit | T value | P |
| NLR | 0.70 | 0.11 | 0.48 | 0.92 | 6.12 | < 0.0001 |
| Week | 0.02 | 0.02 | −0.03 | 0.06 | 0.68 | 0.50 |
| Smoking | 0.01 | 0.25 | −0.47 | 0.49 | 0.20 | 0.95 |
| NLR × Week | −0.09 | 0.03 | −0.15 | −0.04 | −3.37 | 0.001 |
| Note: Number of patients = 28; Number of blood samples = 143 | | | | | | |
| The C/D ratio and NLR were converted to natural logarithms.  Smoking was prohibited during hospitalization (during clozapine titration). | | | | | | |
| C/D, concentration-to-dose; NLR, neutrophil-to-lymphocyte ratio; SE, standard error | | | | | | |
